# A home-based self-directed EEG neurofeedback intervention for people with chronic neuropathic pain following spinal cord injury (the StoPain Trial): description of the intervention

**DOI:** 10.1038/s41393-024-01031-3

**Published:** 2024-09-12

**Authors:** Negin Hesam-Shariati, Lara Alexander, Kevin Yi Chen, Ashley Craig, Paul A. Glare, Mark P. Jensen, Chin-Teng Lin, James H. McAuley, James W. Middleton, G. Lorimer Moseley, Toby Newton-John, Sebastian Restrepo, Ian W. Skinner, Pauline Zahara, Sylvia M. Gustin

**Affiliations:** 1 NeuroRecovery Research Hub, School of Psychology, University of New South Wales, Sydney, NSW Australia; 2 Centre for Pain IMPACT, Neuroscience Research Australia, Sydney, NSW Australia; 3 The Kolling Institute, Faculty of Medicine and Health, University of Sydney, Sydney, NSW Australia; 4 Northern Clinical School, Faculty of Medicine and Health, University of Sydney, Sydney, NSW Australia; 5 Department of Rehabilitation Medicine, University of Washington, Seattle, WA USA; 6 CIBCI Lab, Human-centric Artificial Intelligence Centre, Australian AI Institute, FEIT, University of Technology Sydney, Sydney, NSW Australia; 7 School of Health Sciences, University of New South Wales, Sydney, NSW Australia; 8 IIMPACT in Health, University of South Australia, Kuarna Country, Adelaide, SA Australia; 9 Graduate School of Health, University of Technology Sydney, Sydney, NSW Australia; 10 School of Allied Health Exercise and Sports Sciences, Charles Sturt University, Port Macquarie, NSW Australia

**Keywords:** Chronic pain, Randomized controlled trials, Neuropathic pain

## Abstract

**Study design:**

Randomised controlled trial.

**Objectives:**

The objective is to describe an electroencephalography (EEG) neurofeedback intervention that will be provided in a randomised controlled trial for people with neuropathic pain following spinal cord injury (SCI): the StoPain Trial. In this trial, participants in the treatment group will implement an EEG neurofeedback system as an analgesic intervention at home, while participants in the control group will continue with the treatments available to them in the community.

**Setting:**

University-based study in Sydney, Australia.

**Methods/results:**

This manuscript describes the rationale and components of the EEG neurofeedback intervention designed for individuals with SCI neuropathic pain and intended for home-based implementation. Our report is based on the criteria of the Template for Intervention Description and Replication (TIDieR) checklist, and includes why the efficacy of EEG neurofeedback will be investigated, what will be provided, who will administer it, and how, where, when, and how much the EEG neurofeedback intervention will be administered.

**Conclusions:**

This manuscript provides a detailed description of a complex intervention used in a randomised controlled trial. This description will facilitate the subsequent interpretation of the trial results and allow for the replication of the intervention in clinical practice and future trials.

**Sponsorship:**

Australian Government Medical Research Future Fund (2020 Rare Cancers Rare Diseases and Unmet Needs Scheme: 2006020).

## Introduction

Neuropathic pain develops in almost half of the population with spinal cord injury (SCI) [1, 2]. It is characterised by sensations such as burning, sharp, or shooting at or below the level of injury. The ongoing experience of pain is often accompanied by reduced psychosocial well-being [3] and lowered health-related quality of life [4]. While pharmacological treatments are commonly used to manage neuropathic pain after SCI, the limited efficacy and severe side effects of the available treatments [5–8] highlight the necessity for additional treatment options.

There is preliminary evidence supporting the potential for electroencephalography (EEG) neurofeedback for reducing neuropathic pain after SCI [8–11]. This technique aims to regulate the abnormal electrical brain activity associated with the ongoing experience of pain. Studies indicate that individuals with SCI neuropathic pain often exhibit increased theta frequency and possibly high-beta frequency bands, while showing a decrease in alpha and low-beta frequency bands compared to able-bodied subjects and those with SCI who have no pain [12–14].

In EEG neurofeedback, surface EEG is recorded, processed and presented to participants as a form of positive/negative visual or auditory feedback, all in real-time. This approach aims to help individuals gain control over their abnormal brain activity, ultimately leading to a reduction in pain levels [15]. The existing evidence supporting EEG neurofeedback for neuropathic pain after SCI primarily derives from small-scale, single-arm trials [15–17]. Therefore, conducting a fully-powered randomised controlled trial (RCT) is necessary to evaluate efficacy and document any potential side effects. The StoPain trial will explore a novel in-house-developed EEG neurofeedback intervention for individuals with neuropathic pain following SCI. This RCT will involve 134 participants (Sample size calculation is available in the Supplementary Material), randomly assigned to receive either usual care or usual care along with a 20-day EEG neurofeedback intervention over a four-week period. To be eligible, participants must have a complete or incomplete SCI, and experience persistent neuropathic pain (rated on average as 4 out of 10 or greater on a 0–10 scale) at or below the level of injury for more than three months (Full eligibility criteria is available in the Supplementary Material). The primary outcome in this trial will be pain severity, assessed as the average of four 24-hour recall ratings of pain intensity from the Brief Pain Inventory (BPI) – Pain Severity [18] administered within a 7-day window at each assessment point. The secondary outcomes include pain interference (assessed by the BPI – Pain Interference [18]), sleep disturbance (assessed by the PROMIS sleep disturbance short form [19]), depression (assessed by Patient Health Questionnaire-9 [20]), quality of life (measured by Short-Form survey (SF-36) modified for SCI [21]), and resting-state EEG. Additionally, semi-structured interviews (over the phone or Zoom) will be conducted at post-intervention for participants in the treatment group.

Different neurofeedback techniques have been used to improve chronic pain conditions [22–26]. However, a notable challenge in the StoPain trial involves the type of neurofeedback utilised, where participants are required to develop their own mental strategies to engage with the neurofeedback interface and gain control over their brain rhythms. Another challenge in this trial is providing participants with clear guidance and instructions to facilitate their full participation, enabling them to conduct the neurofeedback sessions at home on their own or conduct the sessions with the assistance of a caregiver if they have tetraplegia. Given the self-directed and home-based nature of this intervention, ensuring oversight of treatment implementation is also essential.

To the best of our knowledge, the StoPain trial is the first RCT incorporating an in-house-developed EEG neurofeedback system, specifically designed for people with SCI neuropathic pain. The aim of this manuscript is to provide a detailed description of the EEG neurofeedback intervention that will be utilised in this trial. The intervention will be outlined in accordance with the six criteria of the Template for Intervention Description and Replication (TIDieR) guidelines [27]. The TIDieR checklist is available in the Supplementary Material. Describing complex interventions is important for the interpretation of the subsequent trial results and enhancing the reproducibility of the intervention in clinical practice and future research [28].

## Methods/results

### Rationale of the intervention

The EEG neurofeedback intervention utilised in the StoPain trial is directed at regulating the abnormal brain rhythms associated with the ongoing experience of neuropathic pain. A recent systematic review on the EEG biomarkers of chronic neuropathic pain identified that EEG bandwidth power was higher in the theta (θ, 4–7 Hz) and high-beta (high-β, 20–30 Hz) bands, but decreased in the high-alpha (high-α, 10–12 Hz) and low-beta (low-β, 13–20 Hz) bands in individuals with both peripheral and central chronic neuropathic pain [14], compared to those without pain. Hence, the neurofeedback training protocol in our intervention will aim to suppress theta (θ, 4–7 Hz) and high-beta (high-β, 20–30 Hz) bands, while reinforcing high-alpha (high-α, 10–12 Hz) and low-beta bands (low-β, 13–15 Hz), which together fall into the sensorimotor rhythms (SMR, 10–15 Hz).

The neurofeedback training protocol will target C3 and C4 electrode sites, as they are located over the sensorimotor cortex, a brain region responsible for integrating sensory inputs, including nociceptive input [29]. Neuropathic pain after SCI is associated with disruptions in normal sensory processing within the brain [30]. Specifically, SCI can lead to cortical reorganisation [31] and altered excitability in the sensorimotor cortex [32], which contribute to the experience of ongoing pain. Additionally, the disruption of communication between the thalamus and sensorimotor cortex, known as thalamocortical dysrhythmia, is a key factor in the development and maintenance of neuropathic pain following SCI [13]. This disruption can alter sensory processing and pain modulation pathways, exacerbating the experience of neuropathic pain.

The meta-analysis findings from a systematic review suggested that EEG neurofeedback might provide clinically meaningful benefits in pain intensity and pain interference for individuals with chronic pain [9]. However, the review also highlights the lack of adequately powered RCTs, particularly for neuropathic pain following SCI. Therefore, while there is room for optimism regarding the analgesic effects of EEG neurofeedback, there remains a need for well-powered RCTs with comprehensive reporting of side effects to evaluate the efficacy of EEG neurofeedback for SCI neuropathic pain.

The primary objective of the StoPain trial is to evaluate the efficacy of a home-based self-directed EEG neurofeedback intervention in reducing pain severity compared to usual care for individuals with neuropathic pain following SCI. The secondary objectives are to explore whether EEG neurofeedback improves psychosocial outcomes, or affects resting-state EEG compared to usual care. Additionally, the acceptability of this home-based self-directed intervention will be evaluated using data from semi-structured interviews with participants in the treatment group.

The StoPain trial follows a parallel design where participants will be randomly assigned to either the treatment or the control condition. The randomisation schedule will be generated by an independent statistician. Participants in the treatment condition will be asked to complete one session of neurofeedback per day for 20 days over a 4-week period. Each session will comprise 5 × 2.5-min rounds of neurofeedback with a break of 30 s between each round. Including set-up time, each session will take up to 30 min. Participants in the control condition can continue with all the treatments normally available to them in the community. This trial will be single-blinded, where an independent statistician, not involved in the trial, will analyse the primary and secondary outcomes at the end of the trial.

For individuals with SCI, daily in-person attendance to neurofeedback sessions in clinics or research laboratories is challenging due to mobility and transportation issues. Additionally, those in remote areas face limited access to pain therapy services. Thus, enhancing access to such interventions for people residing in rural areas and/or with limited physical mobility is crucial. Hence, the in-house-developed EEG neurofeedback system utilised in the StoPain trial is designed to be self-administered by participants in their home environment.

### Development of the intervention

The EEG neurofeedback intervention in the StoPain trial was developed by seven members of the research team (SMG, NH-S, PZ, KC, SR, TN-J and LA), who collectively possess clinical and academic expertise in SCI, chronic pain, as well as biomedical, electrical, and software engineering. During the developmental process, in accordance with the practical guide for consumer involvement in health and medical Research [33], several individuals with SCI neuropathic pain were interviewed and consulted, and their valuable advice and feedback were integrated into the system’s development.

#### Initial consumer involvement

During the initial developmental process, we conducted semi-structured interviews with a consumer group of nine individuals with neuropathic pain following SCI. The interviews commenced with a brief introduction to the rationale behind utilising EEG neurofeedback for neuropathic pain after SCI. This was then followed by a 2-min video demonstrating the EEG neurofeedback system, which was still in development at the time. The video featured a participant putting an EEG headset on and playing our neurofeedback game called the ‘Floating Jellyfish’ [34]. The aim of these interviews was to ascertain consumer needs and attitudes towards EEG neurofeedback as an intervention for neuropathic pain after SCI.

Consumers highlighted several positive aspects of the system, emphasising its usability, autonomy, potential efficacy, and gamification features. For instance, one participant remarked, “*As long as the hands are okay, you can put it on your scalp and use it… I don’t see any issues with me using it*” (58-year-old female), while another noted the simplicity of the game interface, “*I think the look of it [the game] was really simple, where you are only looking at what you really need to look at*” (33-year-old male). Additionally, the sense of autonomy conveyed by the system was valued by consumers, with one stating, “*I like that you can pretty much do it anywhere… I like that it’s not restrictive in that way*” (43-year-old female). The same person appreciated the opportunity to explore different mental strategies: “*It [the game] gives you a bit of flexibility to change your thinking to something that might work better… a lot of the time when you’re doing pain management, it’s very prescriptive*” (43-year-old female). Furthermore, consumers found various game features appealing, “*The music was pleasant… it’s good it wasn’t a tune that is knowable… music can trigger memories, especially for people like me*” (43-year-old female), and “*It’s calming, I don’t know if it was the music, but it was cute how it [the Jellyfish] floated across the screen*” (52-year-old female). One consumer also recognised the potential efficacy of the system, noting, “*I have had a little bit of success already using some mindfulness… so I can see that neuroplasticity and changing the way our brain interprets the pain and relaxes in those chronic states, I can see that would be beneficial*” (43-year-old female). Moreover, participants appreciated the gamification aspect, which provided engagement and a measure of progress: “*This kind of gamification is relatable and approachable… You’ll be going to try and break your high score. So, it kind of makes sense that there’s a measure of how good I’m doing this week*” (34-year-old male). Overall, consumers viewed the system as a relatively easy-to-use, drug-free, and portable tool for pain treatment.

Consumers’ feedback led to adjustments in various aspects of the game. Following input from a 34-year-old male, we made the background colour change in the Jellyfish game less flashy and smoother. This input also influenced the development of the subsequent games. Additionally, although most consumers paid little attention to the point counter, a 32-year-old male recommended to disable it due to distraction. Considering this feedback, along with input from team members who tested the game, we removed the point counter during gameplay and instead implemented a score display after each round of game to enhance motivation. Further, some consumers viewed the music of the game as mere background noise, while others expressed a preference for a mute button or the option to choose their own music. However, due to the controlled nature of the trial, offering music selection was not feasible. Nonetheless, we included instructions advising participants to mute the music if they prefer.

### Key components of the intervention

#### Trial kit

For the StoPain trial, participants will receive a trial kit, containing all the necessary tools for home-based implementation. This kit includes an EEG headset with accompanying ear-clips, a Surface Pro tablet pre-loaded with StoPain software application, a USB connector facilitating Bluetooth connection between the headset and tablet, Potassium Chloride (KCl) salt and a bottle for preparation of KCl solution, sponge electrodes, a small plastic container for storing soaked sponges, a mirror to aid in the headset placement, chargers for both the tablet and the headset, and a detailed manual. A short video of the trial kit can be accessed at https://osf.io/2zxhm/.

#### EEG headset

The EEG headset is a key component of the EEG neurofeedback system, specifically designed and developed for the StoPain trial. Several prototypes of the EEG headset were designed and 3D-printed, utilising OpenBCI (openbci.com) electrodes and biosensing board for EEG recording. These prototypes underwent testing to evaluate comfort, ease-of-use, and signal quality. Twelve able-bodied adults (6 females, 6 males) with varying head circumferences (ranging from 52 to 60 cm) and head shapes (round, flat, etc.) tested the headset prototypes. The aim was to ensure proper electrode contact with the scalp and achieve optimal EEG quality across individuals with different head sizes and shapes. Thus, the headset was designed and developed to be one-size-fits-all, with adjustable sliding ear-pieces for enhanced comfort and usability.

**Fig. 1:**
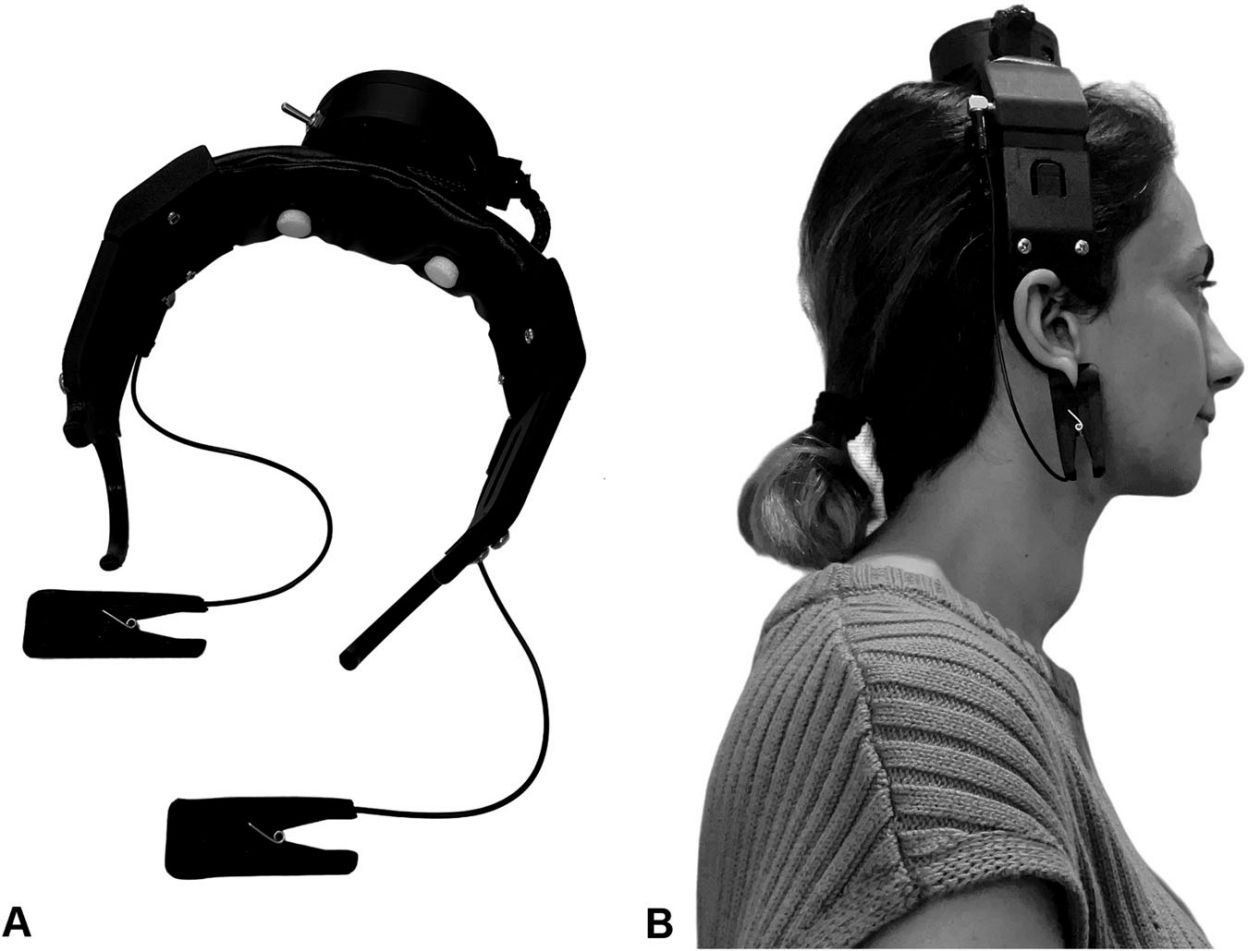
The in-house-developed EEG headset. **A** The headset is equipped with an OpenBCI biosensing board, with two sensors at C3 and C4 electrode sites, and ear-clips serving as the reference electrodes. **B** The placement of the EEG headset on the head.

The end product was a lightweight wireless headset with two sensors at C3 and C4 electrode sites (according to the International 10–20 system), completed with two ear-clips functioning as the reference electrodes (see Fig. 1). C3 and C4 electrode sites cover the sensorimotor region of the brain which is implicated in showing increased theta frequency and decreased alpha frequency power. These changes are believed to underlie SCI neuropathic pain in comparison with both able-bodied individuals and those with SCI but no pain [13, 35]. Sponge electrodes, soaked in a KCl solution are used to establish a low-impedance connection between the electrodes and the scalp, as well as between the ear-clips and earlobes. For setting up the headset, participants will be instructed to pre-soak the sponges in KCl solution, place the sponges in C3 and C4 electrode sites, put the headset on, adjust the ear-pieces, and attach the ear-clips to their earlobes.

#### Software application

A software application (app) was specifically designed and developed for the EEG neurofeedback intervention in the StoPain trial. This app encompasses all instructions and procedural steps essential for conducting home-based sessions as well as the neurofeedback games. After connecting to their home Wi-Fi, inserting a USB connector for Bluetooth connection, and turning the headset on, participants are able to log in to the app using their pre-set participant ID. They will then be guided through a series of prompts in the app to set up the headset, check the electrodes impedance connection, and conduct a resting-state EEG recording before engaging in the neurofeedback games.

**Fig. 2:**
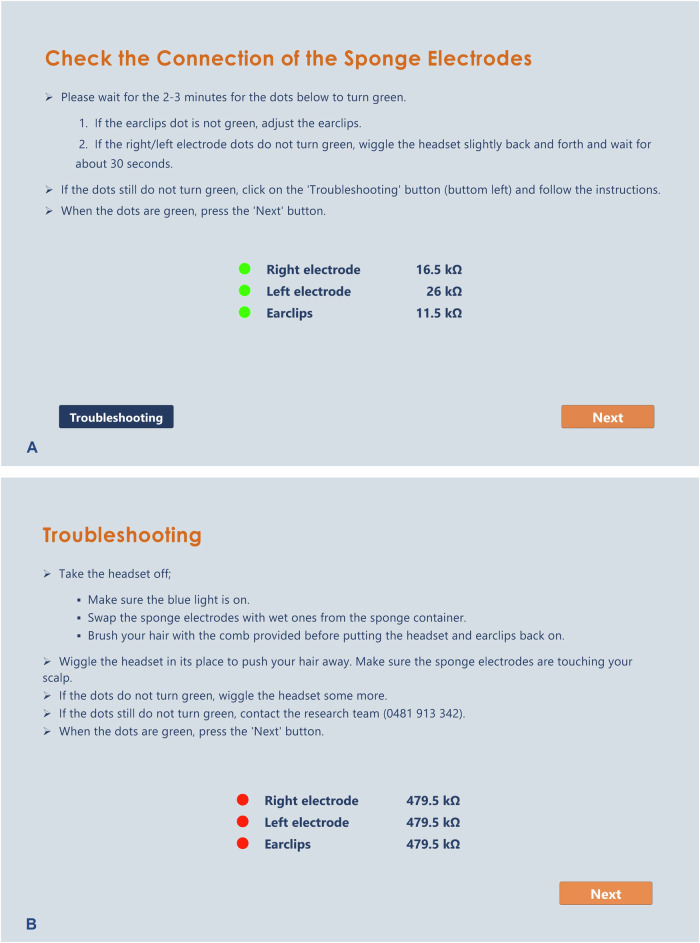
Impedance verification instructions. **A** Prticipants will be instructed to check the electrodes impedance connection. If participants encounter issues to turn the impedance indicators green (< 50 kΩ), they will be guided to press the troubleshooting button, prompting access to the troubleshooting screen. **B** In the troubleshooting screen, they will be led through some simple steps to resolve the issue.

Impedance connection verification is performed automatically through the app. If the impedance levels fall within the accepted range ( < 50 kΩ) participants can proceed to the next step (see Fig. 2A). However, if the impedance levels are over 50 kΩ, participants must go through some simple troubleshooting steps to resolve the issue (see Fig. 2B).

**Fig. 3:**
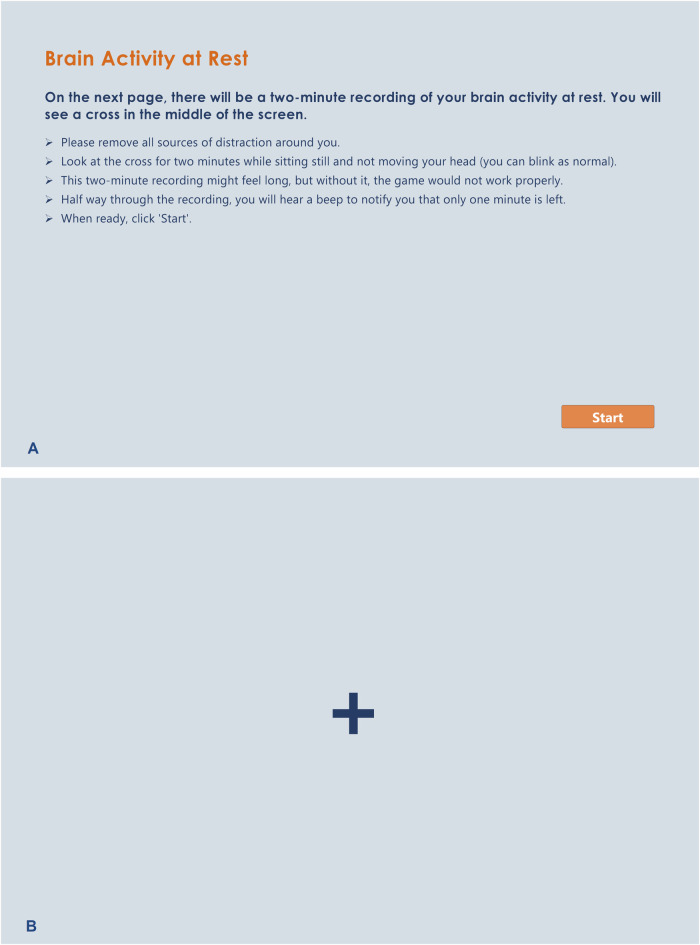
Resting-state EEG instructions. **A** Participants will be directed to look at a fixation cross for a duration of two minutes to record their resting-state EEG. This recording is used to set a threshold for the targeted frequencies during the games. **B** The fixation cross.

During the resting-state EEG recording, participants are instructed to look at a cross positioned in the centre of the screen for a duration of two minutes with minimal movement (see Fig. 3). This recording is used to set a customised baseline for the targeted frequencies (θ, SMR, and high-β) power during the games for each participant. Following this, participants will be instructed to complete five rounds of 2.5-min games with breaks between each round.

**Fig. 4:**
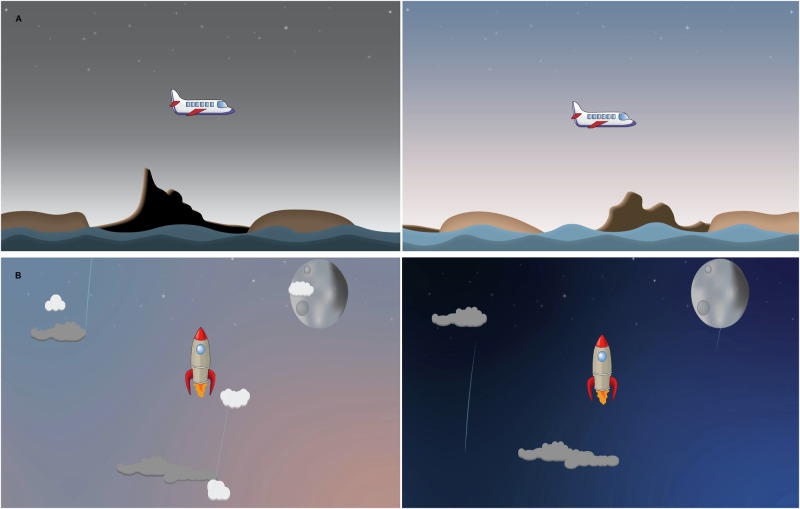
Sample game images. **A** The Plane game: During the Plane game, the plane will fly in the blue sky when participants regulate the targeted frequency bands. **B** The Rocket game: During the Rocket game, the rocket will accelerate through the dark space when participants regulate the targeted frequency bands.

The StoPain software app incorporates four different neurofeedback games, each featuring a unique scenario: a Jellyfish, a Bird, a Plane, and a Rocket (see Fig. 4). To maintain variety and engagement, the app is programmed to introduce one of the games every five sessions (every week) in a particular order to be consistent across participants throughout the intervention period. The games provide visual feedback to participants through changes in the motion of the game character and the background colour, indicating successful regulation of all targeted frequencies (θ, SMR, and high-β). For example, in the Plane game, when the targeted frequency bands are regulated, the plane flies higher in the sky and the background changes to daylight. Similarly, in the Rocket game, successful regulation of the targeted frequency bands makes the rocket to ascend faster into space, with the background turning dark (see Fig. 4). Participants will receive a score for every second that they successfully regulate all three targeted frequencies. These scores will be shown to participants at the end of each round. The within and between-session scores will provide valuable information about the participants’ engagement and progression in regulating their brain rhythms.

**Fig. 5:**
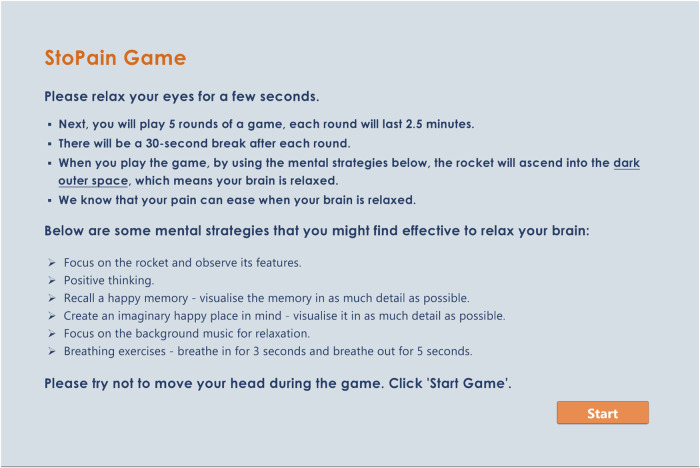
Game instructions. To play the games, participants will be encouraged to use mental strategies that promote a relaxed and mindful state, ones that align all targeted frequency bands in the correct direction.

Participants are encouraged to develop their own mental strategies to interact with the games. While certain strategies (see Fig. 5) will be recommended for participants to experiment during the trial, the aim is for them to explore different strategies and determine which one(s) are most effective for interacting with the games. During the semi-structured interviews at post-intervention, participants will be asked about their developed mental strategies and the one(s) they found most effective for making changes in the visual feedback. Some recommended strategies that may be beneficial include positive thinking [36], focusing on the game features [26, 37], recalling pleasant memories, creating an imaginary happy place, and breathing exercises [38, 39]. Additionally, the games feature relaxing background music intended to help participants become more relaxed [38] and mindful [39]. Participants are advised to adjust the music volume according to their preference, allowing them to increase, decrease, or mute it.

### Consumer feedback integration

#### Trial kit

Two consumers with SCI neuropathic pain were invited to independently conduct a session of EEG neurofeedback using the trial kit and the provided manual (available at https://osf.io/2zxhm/), following a brief introduction on the rationale behind utilising EEG neurofeedback. One consumer (60-year-old male) observed that the positioning of the labels on the contents of the trial kit was not easily visible for individuals in wheelchairs. The labels were then adjusted based on this feedback. The other consumer (57-year-old male) expressed appreciation for the portability of the EEG neurofeedback system, highlighting its suitability for use in various locations.

#### EEG headset

The EEG headset underwent testing with three consumers with SCI neuropathic pain to gather feedback about comfort and ease-of-use. They were provided a brief introduction to the application of the EEG headset, a printed manual, and a short instructional video (available at https://osf.io/2zxhm/) on how to set it up on the head. One consumer (57-year-old male), who had previous experience with the Ultracortex EEG headset from OpenBCI, found our headset impressive, noting that it was much lighter, more comfortable, and better fitted on the head. Additionally, he found the instructional video to be very helpful and straightforward in guiding the setup process. Another consumer (59-year-old male) commented on the grip of the ear-clips, prompting us to upgrade them to be lighter and smoother on the earlobes (see Fig. 1). Building on additional feedback from the same consumer, we further improved the sliding ear-pieces to ensure easier adjustment on the head (see Fig. 1). The third consumer (60-year-old male) highlighted that the headset was comfortable, lightweight, easy to set up, and he hardly noticed wearing it. He recommended clarifying in the manual that the ear-piece loops do not circumnavigate the ears; this adjustment was implemented accordingly.

#### Software application

The StoPain software app was tested with two consumers experiencing SCI neuropathic pain to obtain feedback about its feasibility and clarity. Following a brief introduction, consumers were asked to read a manual detailing the setup process for the tablet, including connecting to Wi-Fi and the EEG headset. Subsequently, they were instructed to follow the prompts on the app to go through the impedance checking, resting-state recording, and five rounds of the Rocket game. Both consumers found the manual and app instructions easy to follow. Additionally, they were able to effectively employ the recommended mental strategies to engage with the game and get scores.

### Participant support and instructions

To facilitate the home-based EEG neurofeedback intervention, participants will be equipped with a comprehensive manual (can be found at https://osf.io/2zxhm/) included in the trial kit, guiding them through the setup process and preliminary preparations such as connecting to their home Wi-Fi and pre-soaking the sponge electrodes. The manual also offers a summary of the in-built instructions featured within the software app, covering tasks like headset setup, impedance checking, and developing mental strategies. Additionally, for the initial two neurofeedback sessions, participants will receive guidance from a member of the research team via the Zoom platform, who will lead them through the entire process and addresses any queries. Access to a laptop or another electronic device will be required for participants to connect with the research team on Zoom.

### Participant oversight and data security

To monitor participants’ adherence and ensure data protection, we have developed an online dashboard hosted on the Amazon Web Services (AWS) lambda platform. Timestamped EEG data from each session will be automatically stored on this secure platform and linked to a participant ID. Additionally, at the beginning of each session, the tablet provided to participants will capture a photo to validate proper placement of the EEG headset. These photos will be deidentified, encrypted, and automatically stored on the secure AWS database, exclusively accessible to the research team. Tablets are authenticated with an API (Application Programming Interface) key for secure interaction with the database. Access to the database is restricted to researchers who have a username, password, and multi-factor authentication credentials for logging in.

Participants’ photos and EEG data will be reviewed by the clinical trial research team after each completed session to ensure correct placement of the EEG headset and to check the quality of the EEG data. Regarding the quality of the EEG data, we will inspect the data for any unexplained artefact or contaminating noise. Continuous monitoring of both EEG data and photos will allow for identification of any potential issues. If issues arise, the research team will promptly contact participants to provide necessary guidance and solutions. Online interactions, such as Zoom calls, will remain available should participants require further assistance.

## Discussion

The forthcoming StoPain trial has the potential to be a milestone as the first randomised controlled trial investigating the analgesic effect of a home-based self-directed EEG neurofeedback intervention for individuals with neuropathic pain following SCI. The trial outcomes aim to provide evidence not only regarding the intervention’s efficacy but also deeper understanding of its acceptability and usability within the SCI neuropathic pain population.

The home-based nature of this EEG neurofeedback intervention offers a number of advantages. First, it holds promise in extending the accessibility and usability of the intervention, particularly benefiting individuals residing in remote areas or experiencing limitations in daily functioning and mobility. Second, once the initial setup is completed, this approach has the potential to reduce the need for extensive clinical resources by enabling individuals to continue their neurofeedback training at home. Third, the inherent flexibility of the home setting may enhance adherence by accommodating daily neurofeedback sessions, thereby overcoming logistical barriers associated with traditional clinical settings. Finally, equipping individuals with the tools to self-administer neurofeedback sessions may foster a sense of self-efficacy/empowerment and active engagement in pain management, potentially enhancing motivation and treatment adherence.

Nevertheless, it is essential to acknowledge and address the challenges inherent in the home-based implementation of this intervention, particularly concerning accurate placement of the headset and susceptibility to artefacts. Environmental factors such as electronic devices and ambient distractions pose potential barriers to the efficacy of the intervention. Mitigating these challenges necessitates guided instructions and sustained motivation strategies to enhance participant engagement, especially when training reaches stagnation.

Overall, this descriptive paper provides details of the EEG neurofeedback intervention in the StoPain trial, which will increase transparency and facilitate a better understanding of the future results of the trial. Ultimately, the findings of this trial will have the potential to inform and shape future research endeavours in the realm of pain management for individuals with SCI neuropathic pain.

## Supplementary information

Supplementary Material
