# Supplementary material for: A home-based self-directed EEG neurofeedback intervention for people with chronic neuropathic pain following spinal cord injury (the StoPain Trial): description of the intervention
Source: Spinal Cord. 2024 Sep 12;62(11):658–66. doi: 10.1038/s41393-024-01031-3 (PMC11549037; doi:10.1038/s41393-024-01031-3)
Supplement: Supplementary file 1 — Supplementary Material [file 41393_2024_1031_MOESM1_ESM.pdf]

Supplementary Materials

A home-based self-directed EEG neurofeedback intervention for people with chronic neuropathic pain following spinal cord injury (the StoPain Trial): description of the intervention

Negin Hesam-Shariati [1, 2], Lara Alexander [1, 2], Kevin Chen [1, 2], Ashley Craig [3], Paul A. Glare [4], Mark P. Jensen [5], Chin-Teng Lin [6], James H. McAuley [2], James W. Middleton [3], G. Lorimer Moseley [7], Toby Newton-John [8], Sebastian Restrepo [1], Ian W. Skinner [8], Pauline Zahara [1, 2], Sylvia M. Gustin [1, 2]

[1] NeuroRecovery Research Hub, School of Psychology, University of New South Wales, Sydney, Australia

[2] Centre for Pain IMPACT, Neuroscience Research Australia, Sydney, Australia

[3] The Kolling Institute, Faculty of Medicine and Health, University of Sydney, Sydney, Australia

[4] Faculty of Medicine and Health, University of Sydney, Sydney, Australia

[5] Department of Rehabilitation Medicine, University of Washington, Seattle, USA

[6] CIBCI Lab, Human-centric Artificial Intelligence Centre, Australian AI Institute, FEIT, University of Technology Sydney, Sydney, Australia

[7] IIMPACT in Health, University of South Australia, Adelaide, Australia

[8] Graduate School of Health, University of Technology Sydney, Sydney, Australia

Corresponding author: Negin Hesam-Shariati, n.hesamshariati@neura.edu.au

CONTENTS:

The TIDieR Checklist ……………………………………………………………..………………. 2
Trial Design ………………………………………………………………………………………… 4
Eligibility Criteria …………………………………………………………………………..…… 5
Sample Size Calculation ……………………………………………………………………..… 5
Randomisation and Blinding ………………………………………………………………..… 5

TIDieR
Template for Intervention Description and Replication

The TIDieR (Template for Intervention Description and Replication) Checklist*

Information to include when describing an intervention and the location of the information.

Item number	Item	Page number **
	**BRIEF NAME**	
**1.**	Provide the name or a phrase that describes the intervention.	1
	**WHY**	
**2.**	Describe any rationale, theory, or goal of the elements essential to the intervention.	3-4
	**WHAT**	
**3.**	Materials: Describe any physical or informational materials used in the intervention, including those provided to participants or used in intervention delivery or in training of intervention providers. Provide information on where the materials can be accessed (e.g., online appendix, URL).	7-8
**4.**	Procedures: Describe each of the procedures, activities, and/or processes used in the intervention, including any enabling or support activities.	8
	**WHO PROVIDED**	
**5.**	For each category of intervention provider (e.g., psychologist, nursing assistant), describe their expertise, background and any specific training given.	4, 5, and 9
	**HOW**	
**6.**	Describe the modes of delivery (e.g., face-to-face or by some other mechanism, such as internet or telephone) of the intervention and whether it was provided individually or in a group.	5 and 9
	**WHERE**	
**7.**	Describe the type(s) of location(s) where the intervention occurred, including any necessary infrastructure or relevant features.	5 and 9

	**WHEN and HOW MUCH**	
**8.**	Describe the number of times the intervention was delivered and over what period of time including the number of sessions, their schedule, and their duration, intensity or dose.	5
	**TAILORING**	
**9.**	If the intervention was planned to be personalised, titrated or adapted, then describe what, why, when, and how.	N/A
	**MODIFICATIONS**	
**10.‡**	If the intervention was modified during the course of the study, describe the changes (what, why, when, and how).	N/A
	**HOW WELL**	
**11.**	Planned: If intervention adherence or fidelity was assessed, describe how and by whom, and if any strategies were used to maintain or improve fidelity, describe them.	10
**12.‡**	Actual: If intervention adherence or fidelity was assessed, describe the extent to which the intervention was delivered as planned.	N/A

** **Authors** - use N/A if an item is not applicable for the intervention being described. **Reviewers** – use '?' if information about the element is not reported/not sufficiently reported.

† If the information is not provided in the primary paper, give details of where this information is available. This may include locations such as a published protocol or other published papers (provide citation details) or a website (provide the URL).

‡ If completing the TIDieR checklist for a protocol, these items are not relevant to the protocol and cannot be described until the study is complete.

* We strongly recommend using this checklist in conjunction with the TIDieR guide (see *BMJ* 2014;348:g1687) which contains an explanation and elaboration for each item.

* The focus of TIDieR is on reporting details of the intervention elements (and where relevant, comparison elements) of a study. Other elements and methodological features of studies are covered by other reporting statements and checklists and have not been duplicated as part of the TIDieR checklist. When a **randomised trial** is being reported, the TIDieR checklist should be used in conjunction with the CONSORT statement (see http://www.consort-statement.org) as an extension of **Item 5 of the CONSORT 2010 Statement.** When a **clinical trial protocol** is being reported, the TIDieR checklist should be used in conjunction with the SPIRIT statement as an extension of **Item 11 of the SPIRIT 2013 Statement** (see http://www.spirit-statement.org). For alternate study designs, TIDieR can be used in conjunction with the appropriate checklist for that study design (see https://www.equator-network.org/).

Trial Design

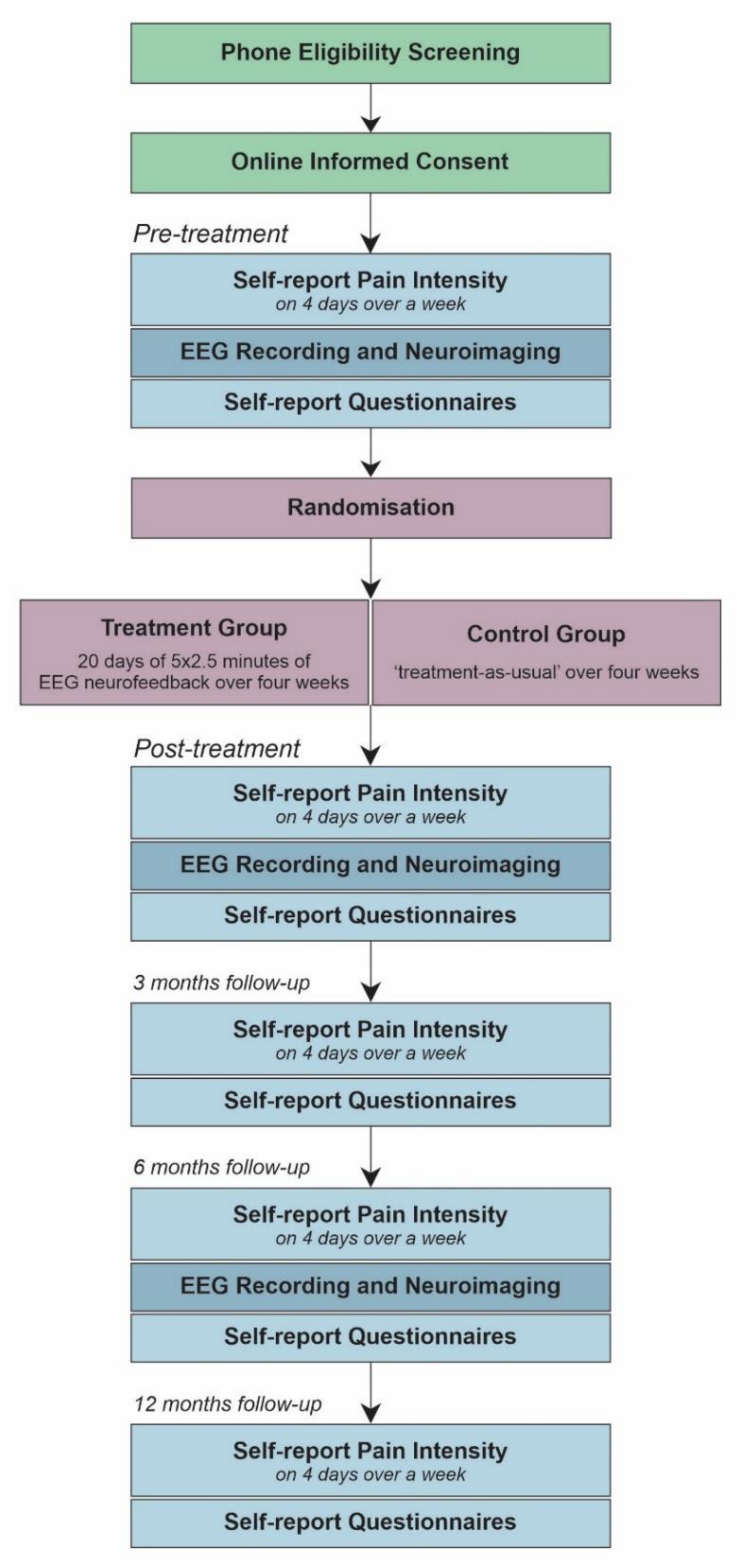

Eligibility Criteria

The inclusion criteria are:

- Having a complete or incomplete cervical, thoracic, or lumbar spinal cord injury,
- Having persistent neuropathic pain at or below the level of injury for more than three months,
- Having an average neuropathic pain intensity of at least 4/10 over the last week,
- Being 18 years or older,
- Residing in Australia,
- Being able to read and understand English, and
- Having the ability to fully participate in the EEG neurofeedback trial (must be able to put on the EEG headset or have a carer to assist).

The exclusion criteria are:

- Having a cardiac pacemaker, cochlear implant, or deep brain stimulation,
- Having neurological disorders with cognitive impairment such as dementia, or a diagnosis of psychiatric disorders such as schizophrenia, and
- Not being able to breathe independently.

Sample Size Calculation

The plan is to enrol 134 individuals with chronic neuropathic pain after SCI to complete the trial. The estimate from the power analysis shows that 116 individuals (58 randomised to Treatment condition and 58 to Control condition) will provide 80% power to detect a 1-point difference in the primary outcome (pain intensity), which is considered to be the minimal clinically important difference between the means of the two groups. We assume a 1.9 standard deviation for each group and a two-tailed Type I error rate of 0.05 to test the difference between means. To account for possible attrition (dropouts before end of the trial) at a rate of 15%, we plan to recruit n = 134 (67 participants in each condition) to achieve 116 (58 participants in each condition). We will include all randomised participants in the analyses (intention-to-treat).

Randomisation and Blinding

Participants (n=134) in this trial will be randomised to either Treatment or Control condition. The random sequence allocation will be conducted in blocks of 6 by an independent statistician using computerised random number generation.

Participants in this trial will be randomised into the treatment vs treatment-as-usual groups, so the clinical trial research team and participants cannot be blinded. However, the randomisation schedule will be generated by an independent statistician who is not involved in the recruitment, treatment, or data collection to minimise risk of bias. The independent statistician, who is blinded to the data collection, will conduct the data analyses at the end of the trial.
